# Does Antiretroviral Therapy Packaging Matter? Perceptions and Preferences of Antiretroviral Therapy Packaging for People Living with HIV in Northern Tanzania

**DOI:** 10.2147/PPA.S238759

**Published:** 2020-01-23

**Authors:** Charles Muiruri, Shelley A Jazowski, Seleman K Semvua, Francis P Karia, Brandon A Knettel, Leah L Zullig, Habib O Ramadhani, Blandina T Mmbaga, John A Bartlett, Hayden B Bosworth

**Affiliations:** 1 Department of Population Health Sciences, Duke University School of Medicine, Durham, NC, USA; 2 Duke Global Health Institute, Duke University, Durham, NC, USA; 3 Department of Medicine, Kilimanjaro Christian Medical University College, Moshi, Tanzania; 4 Department of Health Policy and Management, University of North Carolina at Chapel Hill, Chapel Hill, NC, USA; 5 Center of Innovation to Accelerate Discovery and Practice Transformation (ADAPT), Durham Veterans Affairs Health Care System, Durham, NC, USA; 6 Division of Epidemiology & Prevention, Institute of Human Virology, University of Maryland School of Medicine, Baltimore, MD, USA; 7 Duke Heart Center Nursing Research Program, School of Nursing, Duke University, Durham, NC, USA; 8 Department of Psychiatry and Behavioral Sciences, Duke University, Durham, NC, USA; 9 Division of General Internal Medicine, Duke University, Durham, NC, USA

**Keywords:** antiretroviral therapy packaging, HIV, qualitative research, self-packaging, stigma

## Abstract

**Introduction:**

Despite improvements in treatment (eg, reduction in pill intake), antiretroviral therapy (ART) is dispensed in socially inefficient and uneconomical packaging. To make pills less conspicuous and decrease the risk of being stigmatized, people living with HIV (PLWH) often engage in self-repackaging – the practice of transferring ART from original packaging to alternative containers. This behavior has been associated with ART nonadherence and failure to achieve viral load suppression. While much of the literature on ART packaging has centered around medication adherence, patients stated preferences for ART packaging and packaging attributes that influence the observed ART nonadherence are understudied.

**Methods:**

We conducted a qualitative study to elucidate perceptions of ART packaging among PLWH at two large referral hospitals in Northern Tanzania. Interviews were conducted until thematic saturation was reached. Interviews were audio-recorded, transcribed and coded.

**Results:**

Of the 16 participants whose data were used in the final analysis, a majority were between 36 and 55 years of age (Mean 45.5 years SD: 11.1), had primary-level education (n=11, 68.8%), were self-employed (n=9, 56.3%), reported that they had self-repacked ART (n=14, 88%), and were taking ART for more than 6 years (n=11, 68.8%). Participants identified three attributes of ART packaging that increased anticipated HIV stigma and prompted self-repackaging, including visual identification, bulkiness, and the rattling noise produced by ART pill bottles.

**Conclusion:**

Given the drastic reduction in the number of pills required for HIV treatment, there is an opportunity to not only assess the cost-effectiveness of innovative ART packaging but also evaluate the acceptability of such packaging among PLWH in order to address stigma and improve ART adherence.

## Introduction

At the end of 2017, 36.9 million people were living with HIV worldwide and 21.7 million were taking long-term antiretroviral therapy (ART).^1^ In the past three decades, pharmacological improvements of ART have dramatically reduced the burden of patients’ pill ingestion from approximately 22 pills daily to just a single pill.^2^ Improvements in ART have also reduced medication toxicities and side effects that many patients experienced in the early part of the HIV/AIDS pandemic.^3^

Despite improvements in treatment, HIV remains a highly stigmatized condition throughout much of the world, and there is strong evidence linking stigma to poor clinical outcomes.^3^ The use of ART is closely associated with stigma, particularly for the vast majority of people living with HIV (PLWH) who are asymptomatic, as taking medicine is one of the few outward signs or behaviors that may identify them as being infected.^4^,^5^ Stigma may include internalized feelings of shame and sadness; enacted stigma from others such as gossip, social shunning, or discrimination; or anticipated stigma, including fear that others may learn of their HIV status.^6^

To address concerns about stigma, PLWH often engage in self-repackaging of ART – the practice of discarding pharmacy-issued medication packaging and transferring pills to alternative containers (eg, plastic bags, unmarked pill bottles, or handbags).^7^,^8^ Self-repackaging is often performed to make medication less conspicuous. Self-repackaging has been reported in several studies in sub-Saharan Africa (SSA).^5^,^7^–^10^ In one Ugandan study, adolescent students reported hiding ART in other pill boxes or school bags to avoid unwanted disclosure of their HIV status.^10^ In another Ugandan study, adults living with HIV reported “disguising” ART as a treatment for diarrhea, flu, or vitamin pills.^9^

PLWH may perceive self-repackaging to be harmless or even beneficial by reducing the risk of anticipated stigma. However, among PLWH in Tanzania, we found that self-repackaging of ART was associated with medication nonadherence and failure to achieve viral load suppression.^7^ It is worth noting that since our study was part of a larger study we used quantitative approaches and did not explore the underlying health beliefs of PLWH, the reasons for self-repackaging, and preferences for modifications in or alternative packaging of ART. The authors acknowledged the possibility of unknown or unmeasured confounders influencing the relationship between self-repackaging and ART adherence.

One challenge associated with the practice of self-repackaging is that drugs are purposely packaged by manufacturers to optimize medication stability and therapeutic benefits.^7^,^11^–^13^ For example, packaging provides protection against adverse external conditions, such as light and moisture, which may be particularly important in tropical climates. Packaging also includes printed information intended to assist patients in taking medication as prescribed (eg, instructions about maintaining consistent timing of doses), minimizing side effects (eg, guidance to take with food), expiration dates, and details that may prevent counterfeiting (eg, branding, serial codes).^14^

Pharmaceutical manufacturers are increasingly incorporating packaging features intended to increase adherence.^14^–^17^ For example, “reminder packaging,” such as a pre-filled blister package that clearly labels which day each pill is to be taken, is commonly used for chronic medications (eg, oral contraceptives), in cases of polypharmacy, and has been consistently explored in research studies.^15^ Bosworth et al found that calendared blister packaging was an inexpensive method for improving cholesterol medication adherence among US veterans,^12^ while Schneider et al found that calendared blister packaging was associated with blood pressure control.^18^ By contrast, most ART packaging has not incorporated innovative features; ART are often packed in plastic bottles printed with a “manufacturer’s warning that the product should not be stored outside of the original packaging due to special conditions of conservation.”^16^

To date, findings related to ART packaging have come from broader studies focused on medication adherence and patient-initiated repackaging has received limited attention. Furthermore, patients stated preferences for packaging and also ART packaging attributes that influence the observed ART nonadherence have been understudied. To address this gap in the literature, we conducted a qualitative study among PLWH in Northern Tanzania to understand perceptions of current ART packaging; rationale for self-repackaging and potential associations with stigma and nonadherence; and participants preferences for ART packaging that may inform future innovations.

## Methods

Interview participants were recruited from two care and treatment clinics (CTCs) in the Kilimanjaro Region of Tanzania. Kilimanjaro Christian Medical Centre (KCMC) is a 450-bed zonal referral hospital and Mawenzi Regional Referral Hospital (MRRH) is a 300-bed regional referral hospital, and both of their clinics provide care for over 5,000 PLWH. Pharmacies at both CTCs provide ART in boxes printed with the name of the medication, information about prescribed use, and the expiration date. Each box contains a single pill bottle printed with the aforementioned information and each bottle contains a 30-day supply of pills.

PLWH were eligible to participate if they were at least 18 years of age, fluent in Kiswahili, capable of providing written informed consent, and were receiving HIV care (including receipt of ART prescriptions from CTC-based pharmacies) at one of the two CTCs for at least 12 months. Whether one received care at the two CTCs was confirmed through medical and pharmacy records. We used purposive sampling to recruit participants from the two CTCs. During regular clinic appointments, research nurses informed eligible participants of the study and informed consent was obtained from those agreeing to participate. Interviews were conducted in Kiswahili by trained research nurses and lasted an average of 45 mins. Interviews were recorded, transcribed, and translated into English. Participants were offered remuneration to assist with travel expenses (to/from CTC) at an average of 10,000 Tanzanian shillings (equivalent to approximately $4.35 U.S.).

### Instrument

The semi-structured interview guide was developed through a team-based approach using questions and prompts derived from our previously published work in the region.^7^ The study team also received feedback on the interview guide from a local community advisory board (CAB) on HIV-associated projects and initiatives made up of PLWH, HIV providers, Moshi municipality residents, and other stakeholders. After incorporating CAB feedback, the interview guide was further refined. In order to improve the interview guide by refining the interview questions, we administered the interview guide to nine participants from the KCMC and Mawenzi. The data from the pilot phase were not included in the final analysis. The final interview guide contained questions examining perceptions of ART packaging, reasons for self-repackaging, and recommendations for changes in or alternative ART packaging.

### Data Analysis

Qualitative analysis of the interview transcripts was completed using principles of grounded theory until thematic saturation was achieved. Guest et al proposes that about 12 participants are sufficient to achieve saturation.^19^ Using NVivo software, three authors (SJ, FK, and SS) independently coded five transcripts to identify categories and codes that would later be used to develop a codebook. SJ, FK, and SS then compared the codes, re-read transcripts to evaluate selected codes, and identified higher order themes by integrating conceptually related codes and other emerging themes. Coders then independently coded the remaining transcripts and continually checked for consistency in applying the codebook through discussion and reconciliation. Throughout this process, CM discussed any disagreements to arrive at a consensus among the three coders.

### Ethical Considerations

Informed consent was obtained from all participants included in this study. Participants also provided consent to allow access to their medical records in order to obtain demographic and clinical characteristics This study received ethical approval from the institutional review boards at Duke University, Kilimanjaro Christian Medical College, and the Tanzanian National Institute for Medical Research.

## Results

A total of 16 participants (100% response rate) were approached and consented to participate in the study. Themes saturation was reached at 16 participants. Of the 16 participants, ages ranged between 21 and 64 years (mean age=45.5; SD=11.1 years), the majority of participants had primary-level education (n=11, 68.8%), were self-employed (n=9, 56.3%), reported that they had self-repacked ART (n=14, 88%); had achieved viral suppression (n=11, 67%) and had taken ART for more than 6 years (n=11, 68.8%). Additionally, half of the participants were female and lived less than 11 km from their CTC (Table 1).

**Table 1:** Characteristics of Qualitative Interview Participants

	Participants (N=16)
Demographics	
Age (N, %)^a^	
18–25	2 (13)
26–35	0 (0.00)
36–45	5 (31.3)
46–55	6 (37.5)
56–65	2 (12.5)
≥66	1 (6.3)
Ever self-repacked ART	
Yes	14 (88)
No	2 (12)
Sex (N, %)	
Female	8 (50.00)
Male	8 (50.00)
Marital status (N, %)^b^	
Married	4 (25.00)
Previously married	7 (43.75)
Never married	5 (31.25)
Education (N, %)	
Primary	11 (68.75)
Secondary	5 (31.25)
Occupation (N, %)	
Employed	3 (18.75)
Self-employed	9 (56.25)
Unemployed	4 (25.00)
Distance to clinic (N, %)^c^	
0.0–10.9 km	8 (50.00)
11.0–20.9 km	3 (18.75)
21.0–30.9 km	2 (12.50)
31.0–40.9 km	1 (6.20)
≥41.0 km	2 (12.50)
Clinical Characteristics	
ART duration (N, %)	
1–2 years	2 (12.50)
3–5 years	3 (18.75)
≥6 years	11 (68.75)
Viral load suppression^d^	
Achieved suppression	11 (68.75)
Failure to achieve suppression	1 (6.25)
Missing	4 (25.00)

**Notes:** Demographic and clinical characteristics were self-reported or pulled from medical records. ^a^Age is an estimate based on participants’ recollection of their date of birth. ^b^The Married category includes participants that reported being married or cohabitating and the Previously Married category includes participants that reported being separated, divorced, or widowed. ^c^Distance to clinic is defined as the travel distance from the participant’s residence to the HIV care and treatment center (CTC). ^d^Failure to achieve viral load suppression is defined as HIV RNA level ≥400 copies/mL.

**Abbreviations:** ART, antiretroviral therapy; km, kilometers.

Common themes identified included the reasons for self-repackaging, perceived benefits and challenges of current ART packaging, and recommendations for improving ART packaging moving forward (Table 2).

**Table 2:** Summary of Qualitative Themes Related to Self-Repackaging, Current Benefits of Antiretroviral Therapy (ART) Packaging, and Proposed Modifications to ART Packaging

Themes and Sub-themes	Participants	Illustrative Quotes
Reasons for self-repackaging	14	
Challenges with current packaging	14	
Easily identifiable	14	“I put them [ARTs] in the plastic bag before putting them in the handbag … because I am worried everybody knows the medicines.”
Size/bulkiness	11	“I throw away the box because I think it’s too big and my handbag is small. If I put the medicines with the box there won’t be sufficient space in my handbag.”
Rattling noise	10	“The only problem I see with the container is the noise made by the medicines, which everyone can hear. It makes me feel uncomfortable.”
Imitate observed self-repackaging behavior	2	“I usually remove the box and go with the bottle just like the way I see other people doing.”
Relationship between self-repackaging and perceived adherence^a^	7	
Relationship	6	“If someone is repacking the medicine, they might forget to take them because they might not remember where they kept the medicine, or they might be afraid of taking them when they are with other people.”
No relationship	4	“There is no connection, because there is a special time to take the medicines. Carrying is different from taking them, plus you take the medicines when you reach home.”
Benefits of current packaging	13	
Maintain ART effectiveness	13	“ … the bottle is sealed; this means the medicines are well-packed and no water or air can go inside. Thus, they [ARTs] cannot be easily damaged.”
Usage instructions/ART information	2	“I like to take the medicines in their original box, because I am interested to know about the expiry date.”
Influence medication taking	1	“I think when the medicines are in the bottle it becomes easy to remember taking them.”
Recommendations for patient-centered packaging	13	
Modify current packaging	9	“If it’s possible, can they put something like cotton [in the bottle] so the medicines will not make noise.”
Alternative packaging	6	“I believe if the medicines were blister packed it could make it much easier to take the medicines … it would help those who have no pouch; you can just take them home holding them in your hands.”
Keep current packaging	4	“I think they [ARTs] are well-packed; there is no need to change [the packaging].”

**Notes:** ^a^Participants often provided scenarios where self-repackaging could or could not be related to ART adherence (eg, self-determined patients could be adherent, but other patients may get confused by or forget to take repackaged ART).

**Abbreviation:** ART, antiretroviral therapy.

### Perceived Challenges with ART Packaging and Self-Repackaging

Participants universally acknowledged that repackaging of ART was a common practice among PLWH, and that many individuals would immediately pause outside of the clinic to transfer their medication to other containers. The most commonly cited reasons for repackaging were to hide the medication or make them visually unidentifiable as ART. Participants stated that the manufacturer’s packaging was too visually distinctive and easily recognizable. As a 47-year-old female participant shared, “It is easy for people to recognize the ARTs when they are in their boxes. Even a child can recognize them.”

Aside from concerns about others visually identifying the medication as ART, some participants noted that the packaging was too bulky, so they would transfer the pills to a smaller pouch (eg, handbag) that they could carry without drawing attention to them. Some participants stated that they used plastic bags to reduce the noise produced from rattling pills inside the manufacturer-provided bottles. For example, as a 75-year-old male participant shared, “if the medicines are in the bottle, it’s a normal thing to produce some kind of noise and this is not for ARTs only, it’s for all other types of medicines.”

Participants readily attached the practice of repackaging to the internalized stigma associated with their HIV, as well as anticipated stigma from others. One 75-year-old male participant shared, “Some of us stigmatize ourselves, thinking that if people will see us carrying the medicines in the box, they will know that we have HIV.” Another 60-year-old female participant spoke about her concerns regarding stigma from others, “If people see [the pills], they will not respect the person who takes the medicines because they will know your status.” The necessity of repackaging was also commonly described as being dependent on the context of the patients’ lives and their perceptions of whether others would be accepting of their HIV status. As one 47-year-old female participant commented, “Maybe they are afraid to be stigmatized. You know people live in different circumstances, so it depends on the community that the person lives in, and how they perceive the problem.”

Anticipated stigma may be stronger among PLWH who were recently diagnosed and who have not yet disclosed their HIV status.

There are some patients who don’t like to be seen by others; they hide themselves even while at the clinic. They hide because they are afraid to be seen by the people they know, especially if it’s their first time. (42-year-old female participant)

### Perceived Benefits of Current ART Packaging

When asked whether the manufacturer-provided packaging was advantageous, a majority of participants felt that the packaging maintained the integrity of the medication and its effectiveness. Participants commonly noted that the manufacturer-provided bottles sealed tightly, preventing the pills from being exposed to moisture or contaminants. As one 75-year-old male participant stated, repackaging

can affect the quality of the medicines and the health of the person who takes the medicines, because if the medicines have been repacked and are not kept in a proper bottle, that can allow water or air to come into contact with the medicines.

In addition, some participants felt that the bottle kept the pills secure and prevented them from getting lost or damaged. A small number of participants also indicated that they liked that the current packaging had clearly printed expiration dates and usage instructions, and that the pill bottle acted as a visual reminder to take their medication.

Several participants noted that clinic staff had specifically warned them against repackaging, which led them to discontinue the practice: “The health workers advise us not to repack the medicines because, if we do so, we will make the medicines be less efficient” (50-year-old male participant). However, despite these warnings from healthcare providers, most participants stated the practice of repackaging remained common and ultimately was the patient’s choice. For example, one 60-year-old female participant shared,

You know we are human beings and we are all different. People do different things for different reasons, but we are all adults. We were told the disadvantages of repacking the medicines and we understand them, but still people repack. What can be done then?

### Relationship Between Self-Repackaging and Perceptions of ART Adherence

When asked whether self-repackaging of medications might influence adherence, several participants agreed that the two might be related. For example, one 25-year-old male participant cited that for patients who take more than one medication, repackaging may lead to mislabeling of or confusion about the pills: “There are patients who are supposed to take two drugs in a day, morning and evening. So, if you repack, how are you going to remember which ones to take?”

Many participants minimized the possible connections between self-repackaging and adherence, instead emphasizing the patient’s self-determination as the key factor to adherence. A 47-year-old male participant shared,

There is not any relationship between the way the medicines are packed and taking them. If someone commits to taking the medicines as prescribed, it doesn’t matter how the medicines are packed.

However, several participants felt there was a strong connection among the concepts of stigma, repackaging, and adherence, indicating that the same stigma that leads to self-repackaging may also influence the participant’s adherence:

If the person is repacking the medicines because he is afraid that other people will know that he is having HIV, this can affect the way he takes the medicines. Because if, for example, it’s time to take the medicines and there are people present, this person will be afraid to take them because he will be thinking the people, he is sitting with will know that he has HIV. In this way he will not be taking the medicines as prescribed. (75-year-old male participant)

### Recommendations for Patient-Centered ART Packaging

Participants were asked how the manufacturer’s packaging should be changed to meet their needs. Nearly all participants indicated that they would prefer to receive the pill bottle only, because the box is bulky and easily identifiable. One 50-year-old male participant stated, “I think they should remove the box because of its size. If they remove the box, the bottle will not be easily seen, especially for men who like to put the medicines in their trouser pockets.” Aside from removing the box entirely, participants often indicated the box should be smaller and more nondescript so that others cannot easily identify the medication at first sight.

Participants shared that many PLWH immediately discarded the box in the trash bin upon leaving the clinic. Several noted that they started this practice after observing others at the clinic doing the same: “When I came here the first time, I saw other people removing the box and I decided to do the same” (47-year-old male participant). By contrast, some participants described the inconvenience of having to discard the box once they reached home. For example, one 25-year-old male participant described having to burn the box and keeping the pill bottle after his clinic appointment so that others in his family would not see it.

Although perceived as more acceptable than the box, several participants also disliked the pill bottle, saying it was too large, noisy, and conspicuous: “I think the major reason is the size of the bottle. Sometimes when you walk the medicines will make noise, so you will be afraid because you haven’t accepted your [HIV diagnosis]” (44-year-old male participant). Due to these concerns, participants suggested either packaging ART in smaller containers or including material in the pill bottles (eg, cotton) to stifle or prevent the rattling noise.

Some participants felt it would be ideal if the pills were given in a “small pouch” which would be easy to carry in a pocket or handbag without rattling. One 37-year-old female participant raised the possibility of monthly ART injections, stating this would allow them to receive their medication at the clinic and eliminate the risk of unwanted disclosures in their daily lives. Many participants had encountered blister packaging when taking other types of medication and felt that this type of packaging would be much more desirable than the current bottles. As a 44-year-old male participant noted,

I would suggest the medicines be given in blister packs. Blister packs are transparent, but they are well packed. This will make them easy to carry and patients will feel comfortable to put them in their bag because they do not make noise.

## Discussion

Through interviews with PLWH in Northern Tanzania, we explored and identified several themes related to ART self-repackaging, including perceptions of current ART packaging, implications for stigma and ART adherence, reasons for self-repackaging, and recommendations for alternative ART packaging. Participants identified several attributes of current ART packaging that were related to stigma – visual identification, bulkiness, and the rattling noise produced by the pill bottles. As a result, PLWH commonly self-repackaged their ART into less conspicuous containers, despite guidance that repackaging could negatively influence medication effectiveness. The main strength of this study and contribution to current knowledge is the illumination of PLWH lived experiences with ART packaging. Since our previous published study lacked context and mechanisms that explain the observed poor outcomes.^7^ The results of this study elucidated contextual factors that can inform targeted interventions in order to improve clinical outcomes.

For many pharmaceutical products, visually identifiable packaging is desirable (eg, decreases the risk of counterfeiting),^14^ and design elements are used as a marketing strategy aimed at influencing consumers’ purchasing decisions (eg, customer-friendly packaging equates to consumer loyalty).^20^,^21^ However, for PLWH in this study, the recognizable packaging of ART (eg, bulky packaging, rattling pills) was perceived as an unwanted contributor to indirect HIV disclosures and subsequent enacted stigma. Fear of stigma and its adverse consequences (eg, discrimination, violence, or abandonment)^22^,^23^ influences the adoption of potentially negative health behaviors, such as self-repackaging of ART, pointing to an unmet need for counseling and support programs. For example, future interventions should consider incorporating educational sessions on self-repackaging of ART and the associated negative clinical outcomes into other current counseling activities, including those focused on HIV disclosures and stigma reduction.^23^,^24^

Several studies have indicated an association between self-repackaging and ART nonadherence.^7^,^25^ Therefore, future interventions should focus on designing ART packaging that suits the preferences of PLWH, addresses the drivers of self-repackaging, and maximizes ART adherence. Based on the preferences of participants in this study, ART packaging should be easy to carry (eg, fit in trouser pockets), less conspicuous (eg, produce no rattling noise), not easily recognized by bystanders, and maintain the integrity of the medication. Calendared blister packaging, which meets the aforementioned criteria, has been associated with improved chronic medication adherence and may serve to further support ART adherence.^12^,^26^,^27^ In fact, several participants in this study suggested blister packaging as a welcome alternative to current ART packaging. Given the drastic reduction in the number of pills required for HIV treatment, there is an opportunity to not only assess the cost-effectiveness of innovative ART packaging but also evaluate the acceptability of such packaging among PLWH. These innovations should also incorporate designs that would protect the ARTs from high temperature and moisture in order to reap maximum benefit from adherence.^13^

These findings should be interpreted in light of the study’s limitations. We sought to manage social desirability bias by reassuring participants that their responses would have no impact on their clinical care; however, participants may still have been inclined to respond in a positive manner. With regard to the generalizability of findings, eligible participants were identified by research nurses, and those who were chosen and elected to participate may not be representative of the entire population of PLWH using the CTCs (eg, may reflect persons who were more engaged with their HIV care or likely to agree to participate). Lastly, we did not seek the perspectives of PLWH who were not currently engaged in HIV care, and a majority of participants had viral loads that were undetectable. Those not engaged in care and those who have detectable viral load may have unique perspectives on how the packaging of ART influences their health-seeking decisions and also adherence. Notwithstanding the limitations, this study contributes to the current knowledge medication packaging by incorporating patients' voices in order to generate a hypothesis for future research.

## Conclusions

In this study, participants acknowledged that ART self-repackaging is a common practice intended to make ART less conspicuous, avoid unwanted HIV disclosures, and prevent HIV stigma. To address these concerns, future research should evaluate the cost-effectiveness of different packaging methods, the acceptability of those methods among PLWH, and their potential to reduce anticipated stigma and positively influence ART adherence.

## Data Sharing Statement

The corresponding author has access to the data and is able to provide them upon request.
